# Performance-Based Evaluation of Healing Efficiency on Mechanical Properties of Self-Healing Cementitious Materials Incorporated with PMMA/Epoxy Microcapsule

**DOI:** 10.3390/polym14122497

**Published:** 2022-06-19

**Authors:** Jun Ren, Birunxuan Liu, Hao Li, Ji Zhang, Haiyan Zhu, Meilin Xiao, Guojian Liu, Shuqiong Luo

**Affiliations:** 1 Urban Construction and Digital City Teaching Experiment Center, School of Architecture and Planning, Yunnan University, Kunming 650550, China; renjunking@aliyun.com (J.R.); brxliu@outlook.com (B.L.); ynlihao77@163.com (H.L.); jizhangxm@163.com (J.Z.); zhu_ch555@163.com (H.Z.); mlxiao@ynu.edu.cn (M.X.); 2 School of Civil Engineering, Suzhou University of Science and Technology, Suzhou 215011, China; liuguojian@usts.edu.cn; 3 Henan Key Laboratory of Materials on Deep-Earth Engineering, School of Materials Science and Engineering, Henan Polytechnic University, Jiaozuo 454003, China

**Keywords:** microcapsule, self-healing performance, response surface methodology, mechanical property, recovery rate, healing rate

## Abstract

In this study, based upon the investigation of its effect on workability and the mechanical property of cementitious materials, the Box–Behnken design was adopted to establish models describing self-healing performance on mechanical properties of cementitious materials with polymethylmethacrylate (PMMA)/epoxy microcapsule in terms of healing rate of peak strength (Y1), the recovery rate of peak strength (Y2), the healing rate of Young’s modulus (Y3), the recovery rate of Young’s modulus (Y4), the healing rate of peak strain (Y5), and recovery rate of peak strain (Y6). This was performed under the influence of the four factors, including microcapsule size (X1), microcapsule content (X2), pre-loading (X3), and curing age (X4). The results showed the four factors significantly affect the healing rate and recovery rate of the peak strength, Young’s modulus, and peak strain, except the healing rate on peak strain. Moreover, the interaction between the factors showed some influence as well. The numerically optimised values of X1, X2, X3, and X4 are 203 nm, 5.59%, 43.56%, and 21 days, respectively, and the self-healing cementitious materials with desirable mechanical characteristics (Y1 63.67%, Y2 145.22%, Y3 40.34%, Y4 132.22%, Y5 27.66%, and Y6 133.84%) with the highest desirability of 0.9050 were obtained. Moreover, the porosity of the specimen confirmed the healing performance of PMMA/epoxy microcapsules in cementitious materials.

## 1. Introduction

The degradation and deterioration of concrete, one of the most widely used construction materials, increases the risk of ensuring the safety and durability of concrete structures [1,2,3,4,5]. With the rapid development in civil engineering, the bionics techniques, particularly the self-healing cementitious materials, have received increasing attention from the scientific and engineering communities due to their automatic healing mechanism without human stimulus [6,7,8,9]. In addition to autogenous healing, the self-healing functionality for cement and concrete can be normally achieved by incorporating the artificial agents, for example, the shape–memory alloy, hollow tubes, and microcapsules with healing agents as core, etc., which can partially or completely repair cracks induced from the damage of concrete autogenously and restore the original functionality of the damaged concrete structures [10,11,12,13,14].

Among various concrete healing technologies, embedment of the microcapsules encapsulated with the healing agent to manufacture the self-healing cementitious materials has been widely accepted to be one of the most efficient approaches [15,16,17]. For this method, the microcapsules containing the healing agent are mixed with the cement. After the cracks have occurred in the hardened cement, the shell of the microcapsule can be ruptured, and the healing agent inside can be released. Then, the released healing agent can react with the matrix and repair the cracks [18,19]. Therefore, based on the concept, the properties of the shell and the carrier of the healing agent are important. According to the research, polymeric shells exhibit better versatility than inorganic shells, because the polymer can be artificially tailored to meet the requirement of cementitious materials [20]. Therefore, the most used shell for manufacturing microcapsules is the thermal-set shell, i.e., the urea-formaldehyde shell [21].

Developed from the well-established urea-formaldehyde shell [22,23], researchers have attempted to propose various novel microcapsules with different types of shells to improve the survivability of the microcapsules during early age and to overcome the low triggering efficiency due to the low sensitivity to crack to be ruptured [20,24]. Recently, a novel type of microcapsule with introduced humidity-responsive switchable mechanical properties of polymeric shells has been proposed [25]. However, the improvement in self-healing technology is still not satisfactory due to its incompatibility with the cementitious matrix.

Borrowing the glass transition theory of polymer-based composites, the authors have proposed the temperature adaptive microcapsule with a polymethylmethacrylate-methacrylate copolymer shell and magnesium oxide-based core by adjusting the ratio between methylmethacrylate and methacrylate [26], and determined its performance in cementitious materials under different mixing temperature [27]. Unfortunately, the temperature adaptive microcapsule is based on a solid core, which demonstrated limited applications in preparing self-healing cementitious materials. The development of a liquid-based core temperature adaptive microcapsule is essential. In the field of polymers, polymethylmethacrylate (PMMA) has been applied to manufacture the microcapsules with a liquid-based core for self-healing polymers [28]. However, those microcapsules, in particular, the microcapsule with pure PMMA shell, have not been developed and utilised in manufacturing self-healing cementitious materials, as the glass transition temperature of PMMA is rather high, (i.e., ~105 °C) [26]. However, the manufacture of pure PMMA shell microcapsules can provide a basic investigation on evaluating the performance of temperature adaptive microcapsules in cementitious materials.

To evaluate the performance of a microcapsule in a self-healing cementitious material, its healing effect on the mechanical property of hardened specimens is important. For all types of microcapsules, current studies mainly focus on the effect of microcapsules themselves on the strengths of self-healing materials [19,29,30]. Moreover, the influence of the external environment and curing regime has also been investigated [31,32]. Regarding trigger efficiency, the effect of different proportions of pre-loading has also been studied [33,34]. However, all those studies are designed based on the influence of a single factor, in which the interaction among the factors has been ignored.

It is well-known that the as a mathematical and statistical approach, response surface methodology (RSM) has been widely applied to investigate the influence of independent variables and the interaction among those independent variables, which has demonstrated a great potential for experimental optimisation, particularly in optimising the rheological parameters, hydration and mechanical properties of cementitious materials [35,36,37,38]. Among different RSM approaches, Box–Behnken design (BBD), a rotatable second-order design developed from the three-level factorial design, is often used as a tool for investigation of the influence of variables, in particular, the cases with interactions among variables, due to fewer testing requirements, which has been applied in cement and concrete research [39]. Therefore, considering the unpredictable interactions among the various variables, the BBD provides the potential to study the self-healing performance of PMMA/epoxy microcapsules in cementitious materials under the synergistic effect of various variables.

Thus, in this study, based on the investigation of workability and mechanical property, the BBD was adopted to observe the mechanical properties of the self-healing performance of epoxy/PMMA in cementitious materials. Four factors, in terms of microcapsule size, microcapsule content, pre-loading and curing age, were investigated. Additionally, six healing effects, including the healing rate of peak strength (%), the recovery rate of peak strength (%), the healing rate of Young’s modulus (%), the recovery rate of Young’s modulus (%), the healing rate of peak strain (%), and the recovery rate of peak strain (%) were considered as the responses to evaluate the performance of PMMA/epoxy microcapsules in self-healing cementitious materials. The BBD design was hence conducted to develop three variables (factors) (*n* = 3) experiment matrix with 29 runs in total. In addition, the analysis of variance (ANOVA) was also conducted to assess the adequacy of the regression models. Finally, the pore structure of three selected specimens was determined via mercury intrusion porosimetry (MIP).

## 2. Materials and Methods

### 2.1. Materials

The AR grade chemicals, including methyl acrylate (MA) for preparing the microcapsules shell, methyl acrylate (MA), 2,2′-Abobis (2-methylpropionitrile) (AIBN), mercaptoacetic (MAA), and dichloromethane (DCM), were used to produce PMMA shell. In addition, sodium dodecyl sulphate (SDS) and cetyltrimethylammonium bromide (CTAB) were applied as emulsifier. The epoxy resin E-44 and butyl glycidyl ether (BGE) were used as microcapsule core and diluent, respectively. All chemicals were purchased and directly used without further purification. The imidazole type MC120D was used as curing agent. For preparing the self-healing cementitious materials, standard P.O 42.5 cement in accordance with GB8076-2008 was supplied by Fushun Aosaier Co., Ltd. (Fushun, China). The physico-chemical property of the cement is reported in Table 1.

### 2.2. Preparation and Characterisation of the Microcapsule

The microcapsules with PMMA shell and epoxy core were synthesized in laboratory. The core materials were prepared by mixing the Epoxy E-51 with BGE [40]. The PMMA shell was first synthesised via free radical polymerisation and the synthesis procedure can be referred to in a previous study [26]. After cooling down, the synthesised polymeric shell solution was then directly used for preparing the microcapsule by following the modified procedure proposed by Navarchian’s group [41,42]. The diluted core materials were then mixed with polymeric shell to prepare the oil phase. The prepared solution was then added to the aqueous phase with 1 wt% of emulsifier at room temperature to mix for one hour to form O/W emulsion. Then, the solution was poured into the same aqueous solution emulsifier under continuous agitation. The microcapsules were obtained after evaporating the DCM solution. To manufacture the particles with different particle sizes, three different rotation speeds, i.e., 200, 400, and 600 rpm, were applied to an oil/water emulsion. The obtained PMMA/epoxy microcapsules were then filtered, washed several times with distilled water, and dried at room temperature. 

**Figure 1:**
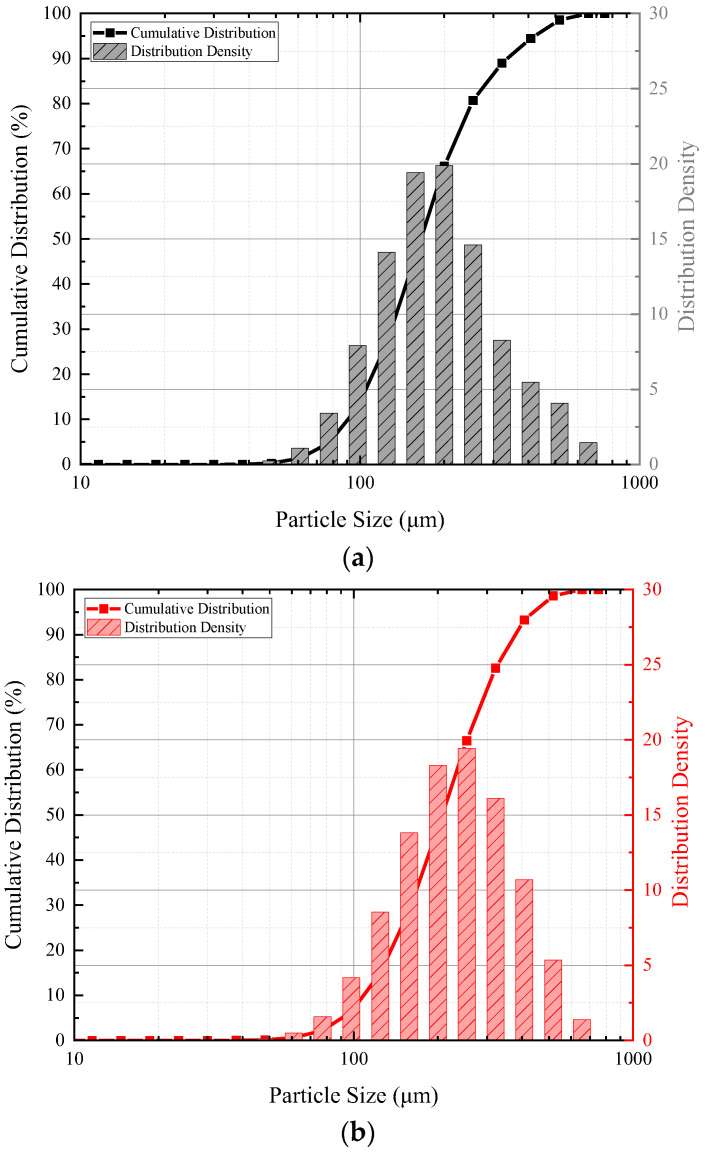

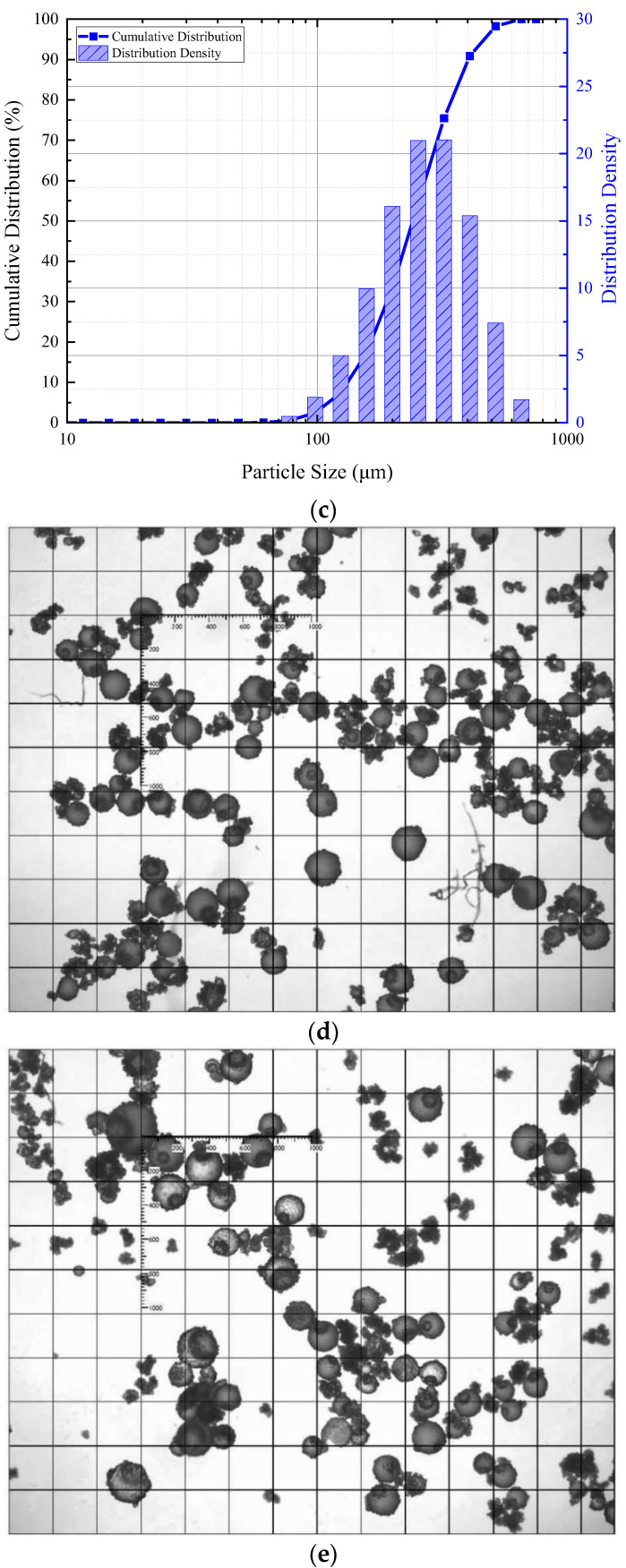

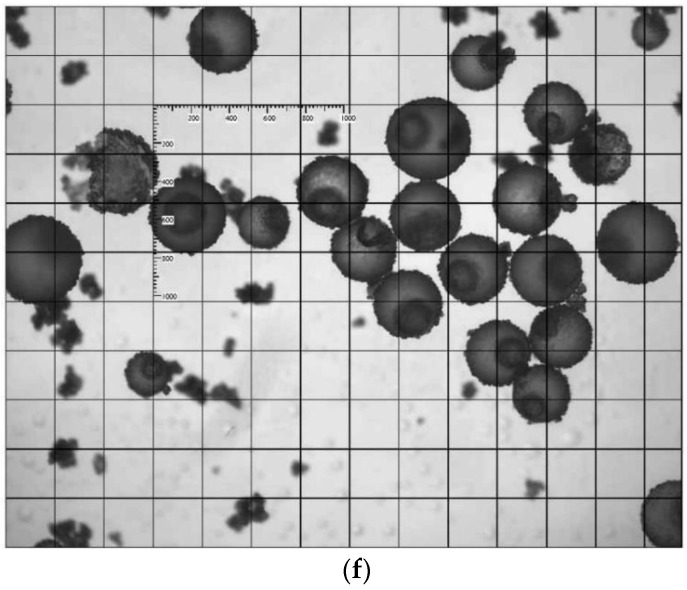
Morphology and particle size distribution of PMMA/epoxy microcapsules under different rotation speeds. (**a**) Particle size distribution and cumulative curve of small microcapsule under 600 rpm; (**b**) particle size distribution and cumulative curve of medium microcapsule under 400 rpm; (**c**) particle size distribution and cumulative curve of large microcapsule under 200 rpm; (**d**) morphology of small microcapsule under 600 rpm; (**e**) morphology of medium microcapsule under 400 rpm; (**f**) morphology of large microcapsule under 200 rpm.

To evaluate the particle size, the size distribution and cumulative curve of three microcapsules were determined via particle size analysis (S3500 Microtrac Inc.) with ethanol as the eluent. The results are presented in Figure 1a–c and details are summarised in Table 2. Moreover, the morphology of the microcapsule was determined by PIP 9.1 Particle Image Processor using an OMEG instrument, which is shown in Figure 1d–e, and the roundness of the microcapsule is presented in Table 2, which suggests that the average sizes of small, medium and large microcapsules were 164.98 µm, 202.38 µm, and 241.16 µm, respectively, and the roundness of three microcapsules were almost the same.

### 2.3. Preparation of Self-Healing Cementitious Materials

The self-healing cementitious paste was prepared with a water to cement ratio (w/c) of 0.29, which was selected based on trial-and-error to achieve standard consistency. The content of microcapsule was fixed at 0%, 2%, 4%, and 6% by weight of the cement. To cure the epoxy, the curing agent MC120D was added, and the weight ratio between epoxy and MC120D was 1:0.4. Considering the requirement of experimental design (which will be discussed later), a total of 10 mixes were prepared and investigated in this paper. Details of mixing proportion are presented in Table 3. For mixing process, the microcapsule and cement were placed in a bowl mixer and pre-mixed at a low-mix speed for 30 s before water was added. After that, the mixture was first stirred at a slow speed for 90 s, then the mixer was paused for 30 s, and the mixture was re-stirred for another 90 s. After mixing, the specimens were removed from moulds after 24 h and cured under standard conduction (20 ± 2 °C, HR > 95%) for 28 days. After that, the surfaces of the specimens were wetted to be ready for testing.

### 2.4. Characterisation and Evaluation of Self-Healing Performance

In accordance with Chinese standard GB/T 8077-2012, the workability of the cement paste was determined via a minislump test using a cone with a top diameter, bottom diameter, and height of 36, 60, and 60 mm, respectively. The average diameter of the spread from the minislump in two perpendicular directions was recorded. The initial minislump measurements were conducted 5 min after mixing.

The specimens with the sizes of 20 × 20 × 20 mm^3^ were prepared for compression test. For different characterisation purposes, the specimens were categorised into three groups, namely Group I, II, and III. Group I was directly subjected to uniaxial compression test until its failure. Group II was preloaded to 20%, 40%, and 60% of the peak strength, and immediately unloaded to achieve damage state. Group III followed the same procedure as Group II to damage state. The damaged specimens were then transferred to a standard curing room (20 ± 2 °C, HR > 95%) and cured for another 7, 14, and 21 days to determine the self-healing performance. The average value of the strength of the specimens with less than 10% difference in each group was recorded. 

To evaluate the effect of microcapsules on the compressive strength of a self-healing cementitious material, the change in the mechanical properties by incorporating the microcapsules was calculated according to Equation (1).

(1) $$R_{f,28d}=\frac{F_{cm}-F_{m}}{F_{m}}\times 100\%$$

where *R_f_* stands for the change rate of mechanical properties, (i.e., peak strength, Young’s modulus, and peak strain) at 28 days by incorporating microcapsules, in which the positive and negative values indicate the increase and decrease in compressive strength, *F_cm_* for the 28 days mechanical properties, (i.e., peak strength, Young’s modulus, and peak strain) of hardened cement paste with microcapsules, *F_c_* for 28 days mechanical properties, (i.e., peak strength, Young’s modulus, and peak strain) of hardened cement without microcapsules.

To evaluate the healing effect, the recovery rate and healing rate were applied. The recovery rate and healing rate of peak strength, Young’s modulus, and peak strain were calculated according to Equations (2)–(7) [34,40,43].

(2) $$\eta_{STR}=\frac{f_{healed}}{f_{initial}}\times 100\%$$

(3) $$\varphi_{STR}=\frac{f_{healed}-f_{damaged}}{f_{damaged}}\times 100\%$$

(4) $$\eta_{E}=\frac{E_{healed}}{E_{Initial}}\times 100\%.\;$$

(5) $$\varphi_{E}=\frac{E_{healed}-E_{damaged}}{E_{damaged}}\times 100\%$$

(6) $$\eta_{\sigma \varepsilon }=\frac{\sigma_{healed}}{\sigma_{Initial}}\times 100\%$$

(7) $$\varphi_{\sigma \varepsilon }=\frac{\varepsilon_{damaged}-\varepsilon_{healed}}{\varepsilon_{damaged}}\times 100\%$$

where $\eta_{STR}$ stands for the recovery rate of peak strength, $\varphi_{STR}$ for the healing rate of peak strength, $\eta_{E}$ for the recovery rate of Young’s modulus, $\varphi_{E}$ for the healing rate of Young’s modulus, *η_σε_* for the recovery rate of peak stress, *φ_σε_* for the healing rate of peak strain, *f_healed_* for the peak strength after healing, *f_inital_* for the peak strength of specimens under initial state, *f_damaged_* for the peak strength after pre-loading, *E_healed_* for Young’s modulus after healing, *E_initial_* for Young’s modulus under initial state, *E_damaged_* for Young’s modulus after pre-loading, *ε_healed_* for the peak strain after healing, *ε_initial_* for the peak strain under initial state, *ε_damaged_* for the peak strain after pre-loading.

The pore structure was determined via MIP test by an AutoPore 9500IV porosimeter (Micromeritics, Atlanta, GA, USA) to determine the porosity and pore size distribution of the selected self-healing cementitious materials. The debris from hardened cement paste was collected and vacuum dried with a pump continuously running for 24 h before the test. The diameter of the pore was obtained by the calculation from Washburn equation [44,45,46].

### 2.5. Experimental Design

Considering the fact that both microcapsule property and curing regime can significantly affect the self-healing performance of PMMA/epoxy microcapsule incorporating cementitious materials, as shown in Table 4, four factors, in terms of microcapsule size, microcapsule content, pre-loading and curing age, were examined. For each factor, there are three levels of values investigated, lower, middle, and higher, which are coded as −1, 0, and 1. Five repeating runs were applied at the central point to evaluate the error of the design matrix [47]. Therefore, in total 29 runs were applied, the details of which are presented in Table 5. For the results, seven responses, including compressive strength reduction (%), recovery rate of compressive strength (%), healing rate of compressive strength (%), recovery rate of Young’s modulus (%), healing rate of Young’s modulus (%), recovery rate of peak strain (%), and healing rate of peak strain (%), were selected as the responses to evaluate the performance of PMMA/epoxy microcapsules in a self-healing cementitious materials. The statistical response models were quantitatively measured for mathematical optimisation. The analysis was conducted using the Design-Expert software (State-Ease Inc. Minneapolis, MN, USA).

## 3. Results and Discussion

### 3.1. Effect on Workability

**Figure 2:**
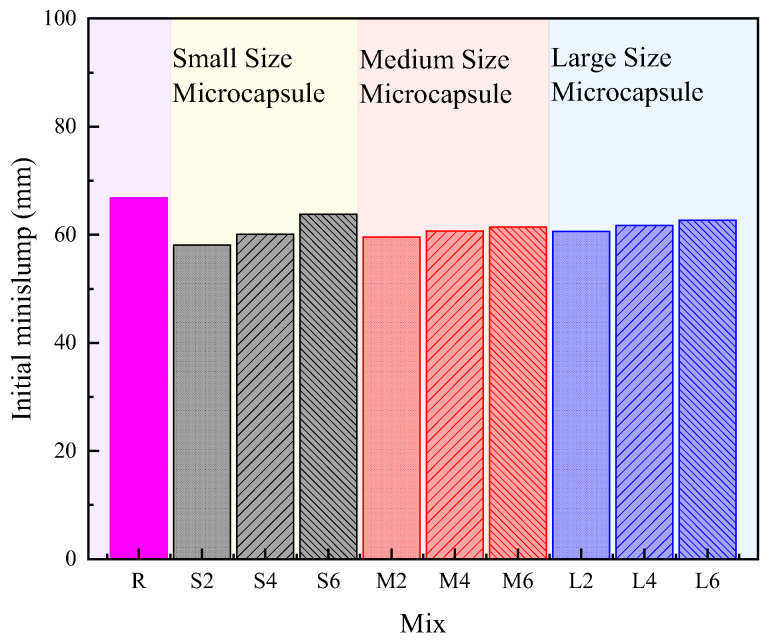
Effect of microcapsule size and content on workability of cementitious materials.

The effect of microcapsule size and content on the workability of cement pastes are presented in Figure 2. It can be clearly seen that the addition of microcapsules slightly decreased the minislump of cement pastes, which was similar to the previous report that the addition of microcapsules with solid MgO core decreased the minislump of cement pastes [27]. This reduction in the initial minislump might be due to the addition of microcapsules that modified the viscosity of the cement pastes by introducing the crowding effect [48,49]. However, compared to the reference [27], the difference between the cement pastes without microcapsules and with microcapsules was not significant. The result could be due to the fact that, in this study, the cement paste under standard consistency was prepared, which provided a limited range for measuring the change in workability. Moreover, although the difference was confined, it was generally shown that the increased microcapsule content increased the workability, in which the microcapsules with small particle sizes were more pronounced. Again, the results for those phenomena could be attributed to the crowding effect of introducing microcapsules into cement pastes.

### 3.2. Effect on Mechanical Property

**Figure 3:**
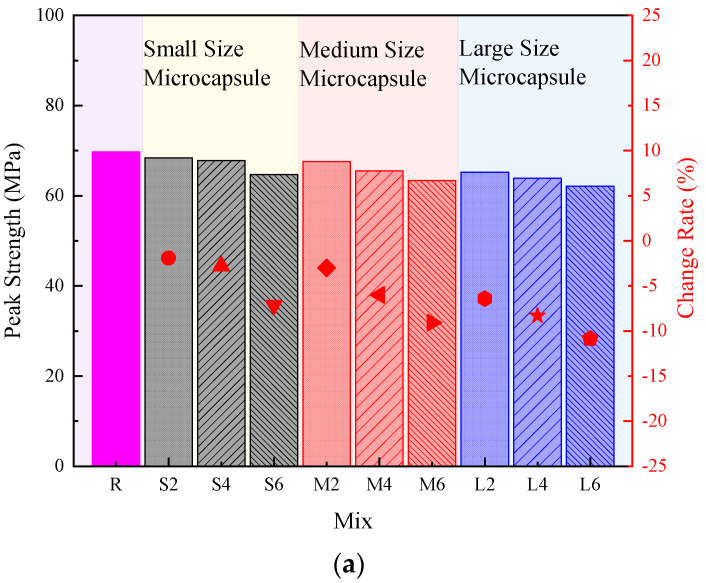

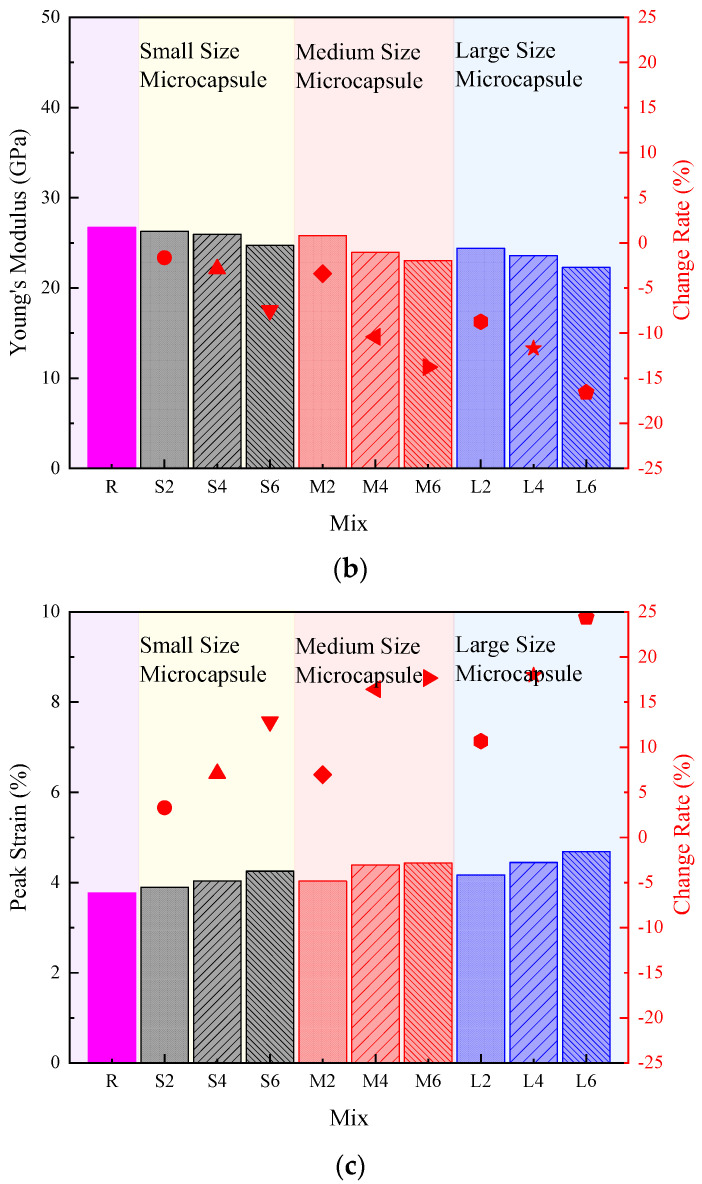
Effect of microcapsule size and content on the mechanical properties and change rate of cementitious materials. (**a**) peak strength; (**b**) Young’s modulus; (**c**) peak strain.

The effect of microcapsule size and content on mechanical properties, in terms of peak strength, Young’s modulus, and peak strain of hardened cement pastes are presented in Figure 3a-c. As shown in the figures, regardless of the size and content of microcapsules, the incorporation of microcapsules decreased the peak strength and Young’s modulus of hardened cement pastes, while it increased the peak strain of the pastes. The trend was similar to the self-healing cementitious materials with UF/epoxy microcapsules [22], which could be attributed to the different properties between the microcapsules and the cementitious matrix and the change in the porosity of cement pastes. It should be noted that Young’s modulus of polymers (in this case, the microcapsule) was less than 10 GPa, while that of cement pastes was in the range between 10–30 GPa [26,50], which leads to the reduction in Young’s modulus of the self-healing cementitious materials. Moreover, the increased peak strain indicated that the microcapsules improve the brittleness of the cementitious materials. This reduction in Young’s modulus and improvement in brittleness could be attributed to the difference in stiffness of microcapsules and cementitious matrix [26,40]. Moreover, the weak interfacial behaviour between microcapsule cores and the cementitious matrix could also contribute to the change in the mechanical properties of self-healing cementitious materials [51].

Obviously, the size of the microcapsule significantly affected the mechanical properties of cement pastes. Generally, the increase in microcapsule size resulted in a lower peak strength, a lower Young’s modulus, and a higher peak strain. For example, when the microcapsule content was fixed as 4% by weight when increasing the microcapsule size from small (165 nm) to large (241 nm), the peak strengths of cement pastes were changed from 67.75 to 63.89 MPa, which was reduced by 5.69%. This could be due to the change in the porosity of the hardened cement paste [33,52]. Similarly, Young’s modulus of cement paste with small and large size microcapsules were 25.96 and 23.60 GPa, which was reduced by 9.90%. However, the peak strain with small and large size microcapsules were 4.03 and 4.45%, which increased by 10.42%.

Moreover, the microcapsule content also played an important role in the mechanical properties of self−healing cementitious materials. As shown in Figure 3a−c, for all types of microcapsules, the increase in microcapsule content decreased the peak strength and Young’s modulus of hardened cement pastes, while the peak strain was increased by increasing the microcapsule content. For example, with the medium size microcapsules, (i.e., 201 nm), compared to the reference sample, (i.e., the mixture without microcapsules), the peak strength of cement with 2%, 4%, and 6% by weight microcapsules were decreased by 3.01%, 6.00%, and 9.06%, respectively, which was similar to the effects of UF/epoxy microcapsules [40]. Meanwhile, Young’s modulus decreased by 3.41%, 10.41%, and 13.76%, respectively, and the peak strain increased by 6.94%, 16.42%, and 16.66%, respectively.

### 3.3. Evaluation of Healing Effect by Experimental Design

#### 3.3.1. Model Adequacy Analysis

The experiment results on the six properties, (i.e., Y1, Y2, Y3, Y4, Y5, and Y6) obtained from all 29 tests are presented in Table 6. Based on the results, the models (based on coded value) of each response were analysed by Design-Expert 12 and are presented in Equations (8)–(13). Obviously, the best fitting surface response models for describing the healing rate of peak strength (Y1), the recovery rate of peak strength (Y2), the healing rate of Young’s modulus (Y3), the recovery rate of Young’s modulus (Y4), and healing rate of peak strain (Y5), are suggested as a quadratic model, whilst the models of the recovery rate of peak strain (Y6), are a linear model. The analysis of variance (ANOVA) was then conducted to assess the significance and adequacy of the six models, which are discussed in separate sections below (Section 3.3.1, Section 3.3.2 and Section 3.3.3). Considering the repeatability of the responses at the central points were normally applied for estimating the error, the six responses at central points were also analysed and the results are presented in Table 6.

(8) $$\begin{array}{cc} Y_{1}=40.91+7.88X_{1}+13.20X_{2}+5.54X_{3}+18.16X_{4}+2.04X_{1}X_{2}-1.73X_{1}X_{3}+ & \\ 4.31X_{1}X_{4}+2.27X_{2}X_{3}+3.09X_{2}X_{4}+2.19X_{3}X_{4}-7.40X_{1}{}^{2}-1.22X_{2}{}^{2}-10.24X_{3}{}^{2}- & \\ 6.15X_{4}{}^{2} & \end{array}$$

(9) $$\begin{array}{cc} Y_{2}=126.57+8.51X_{1}+11.51X_{2}+2.28X_{3}+16.99X_{4}+1.25X_{1}X_{2}-1.14X_{1}X_{3}+ & \\ 4.11X_{1}X_{4}+1.82X_{2}X_{3}+3.51X_{2}X_{4}+1.60X_{3}X_{4}-5.08X_{1}{}^{2}+0.26X_{2}{}^{2}-8.07X_{3}{}^{2}- & \\ 4.19X_{4}{}^{2} & \end{array}$$

(10) $$\begin{array}{cc} Y_{3}=35.10+9.89X_{1}+5.85X_{2}+9.56X_{3}+11.59X_{4}+1.27X_{1}X_{2}+4.02X_{1}X_{3}+ & \\ 4.70X_{1}X_{4}-0.70X_{2}X_{3}+1.93X_{2}X_{4}+3.52X_{3}X_{4}-4.91X_{1}{}^{2}-1.63X_{2}{}^{2}-9.16X_{3}{}^{2}- & \\ 9.09X_{4}{}^{2} & \end{array}$$

(11) $$\begin{array}{cc} Y_{4}=122.72+6.95X_{1}+5.60X_{2}+3.08X_{3}+10.70X_{4}-0.13X_{1}X_{2}+1.91X_{1}X_{3}+ & \\ 3.98X_{1}X_{4}-0.38X_{2}X_{3}+2.42X_{2}X_{4}+2.78X_{3}X_{4}-5.08X_{1}{}^{2}-2.50X_{2}{}^{2}-7.82X_{3}{}^{2}- & \\ 7.81X_{4}{}^{2} & \end{array}$$

(12) $$\begin{array}{cc} Y_{5}=26.24+1.60X_{1}+0.72X_{2}+0.47X_{3}+1.38X_{4}+0.77X_{1}X_{2}-0.15X_{1}X_{3}- & \\ 1.86X_{1}X_{4}-0.65X_{2}X_{3}+1.94X_{2}X_{4}-3.04X_{3}X_{4}-9.37X_{1}{}^{2}-9.99X_{2}{}^{2}-12.25X_{3}{}^{2}+ & \\ 0.12X_{4}{}^{2} & \end{array}$$

(13) $$Y_{6}=118.99+4.76X_{1}+4.06X_{2}+1.18X_{3}+19.06X_{4}$$

#### 3.3.2. Effect of Variables on the Response of the Model

Based on the statistical analysis of the six predicted models, the effects of various variables and their interactions in each predicted model are discussed in this section.

##### Effect on Healing Rate of Peak Strength

**Figure 4:**
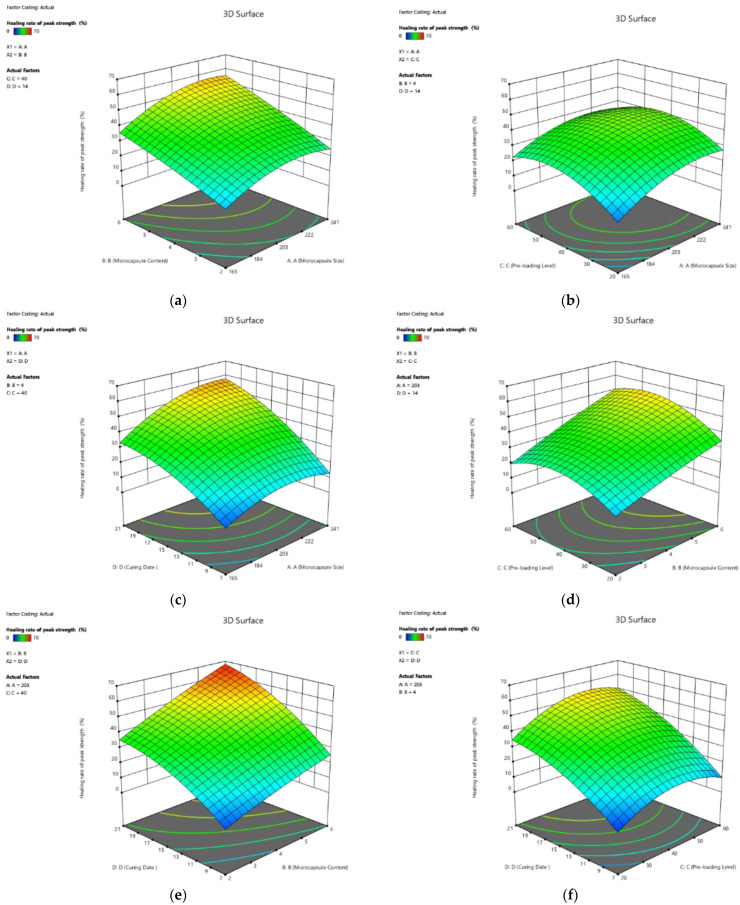
Three-dimension response surface plots of healing rate of peak strength (Y_1_) in relation to: (**a**) microcapsule size (X_1_) and microcapsule content (X_2_); (**b**) microcapsule size (X_1_) and pre-loading level (X_3_); (**c**) microcapsule size (X_1_) and curing age (X_4_); (**d**) microcapsule content (X_2_) and pre-loading level (X_3_); (**e**) microcapsule content (X_2_) and curing age (X_4_); (**f**) pre-loading level (X_3_) and curing age (X_4_).

The 3D response surface plots of the healing rate of the peak strength with the interactive relationship among the four factors are shown in Figure 4a–f. Based on Equation (10), the healing rate of the peak strength response increased with the increase in all four factors, namely, microcapsule size, microcapsule content, pre-loading level, and curing age. Moreover, the higher coefficient of curing age, which was approximately 3.3 times larger than those of pre-loading level, indicates that the curing age significantly affected the peak strength of the pastes. Moreover, the interaction between every two factors showed its influence on the healing rate of peak strength. However, since the value of coefficients was less than that of a single factor, the contribution from those interactions was less.

##### Effect on Recovery Rate of Peak Strength

**Figure 5:**
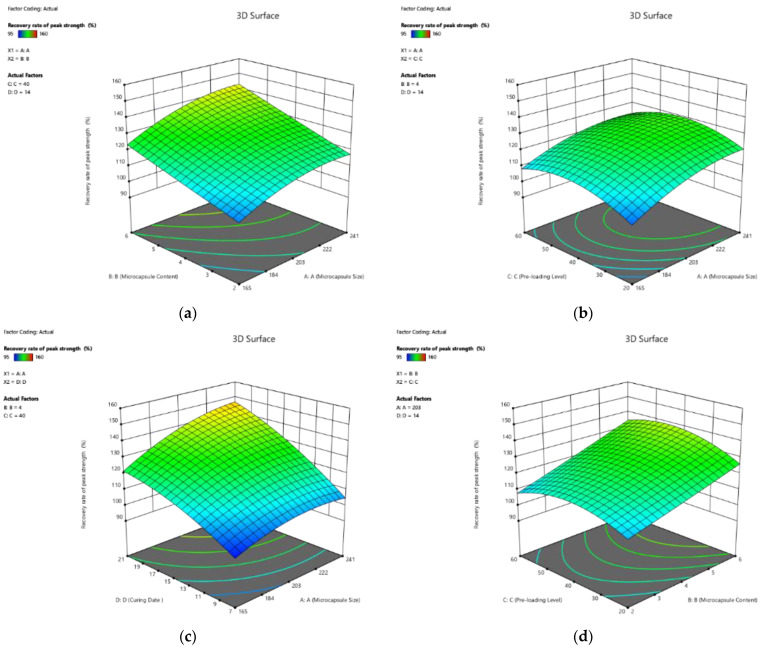

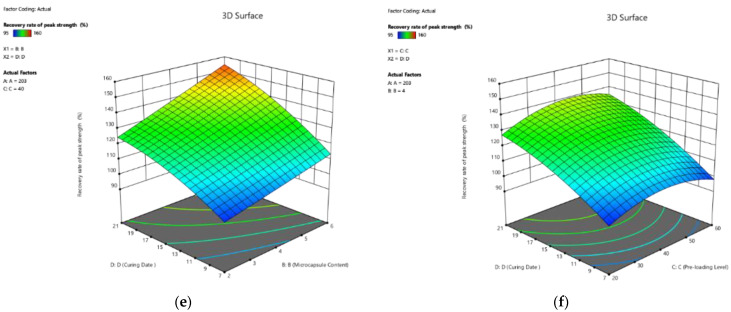
Three-dimension response surface plots of recovery rate of peak strength (Y_2_) in relation to: (**a**) microcapsule size (X_1_) and microcapsule content (X_2_); (**b**) microcapsule size (X_1_) and pre-loading level (X_3_); (**c**) microcapsule size (X_1_) and curing age (X_4_); (**d**) microcapsule content (X_2_) and pre-loading level (X_3_); (**e**) microcapsule content (X_2_) and curing age (X_4_); (**f**) pre-loading level (X_3_) and curing age (X_4_).

The 3D response surface plots, visualising the effect of the four factors on the recovery rate of peak strength of self-healing cement pastes, are plotted in Figure 5. Similar to the influence on the healing rate, the recovery rate of peak strength increased with the increase in all four factors. Moreover, the influences in the order of decreasing magnitude are curing age, microcapsule content, microcapsule size, and pre-loading level. For example, the coefficient of curing age showed an approximately 7.45 times greater effect on increasing the recovery rate than the pre-loading level (16.99 vs. 2.28 in Equation (11)).

##### Effect on Healing Rate of Young’s Modulus

**Figure 6:**
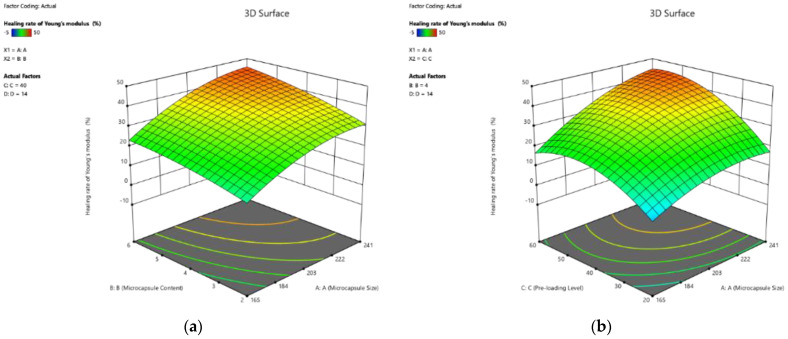

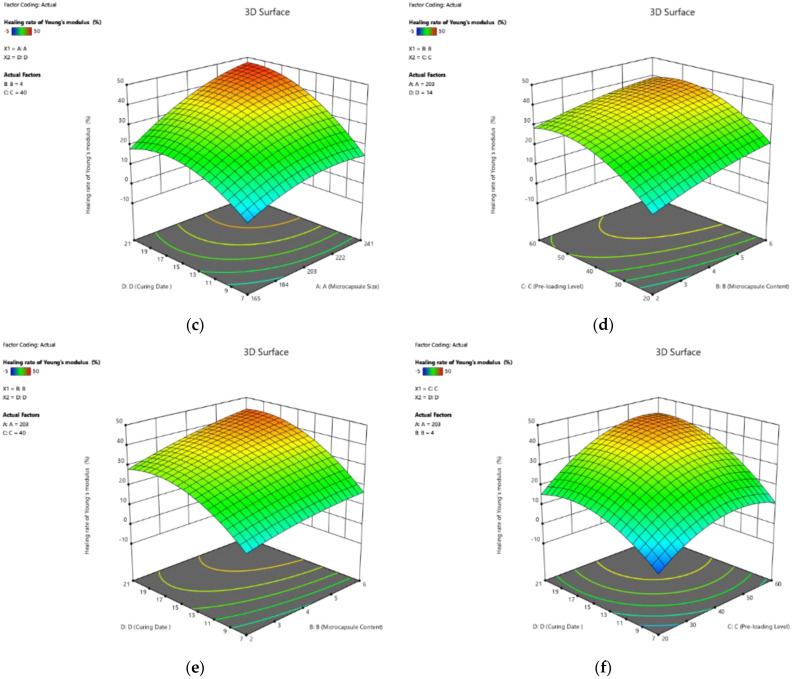
Three-dimension response surface plots of healing rate of Young’s modulus (Y_3_) in relation to: (**a**) microcapsule size (X_1_) and microcapsule content (X_2_); (**b**) microcapsule size (X_1_) and pre-loading level (X_3_); (**c**) microcapsule size (X_1_) and curing age (X_4_); (**d**) microcapsule content (X_2_) and pre-loading level (X_3_); (**e**) microcapsule content (X_2_) and curing age (X_4_); (**f**) pre-loading level (X_3_) and curing age (X_4_).

The 3D response surface plots, visualizing the effect of the four factors on the healing rate of Young’s modulus of cement pastes, are plotted as the response surfaces in Figure 6. As shown in Equation (12), the healing rate of Young’s modulus increased with the increase in the four factors. Moreover, the influences in the order of decreasing magnitude are curing age, microcapsule size, pre-loading level, and microcapsule. However, the difference in contribution to the healing rate was not obvious. Similar to its influence on peak strength, the interaction between the two factors was similar to that of a single factor.

##### Effect on Recovery Rate of Young’s Modulus

**Figure 7:**
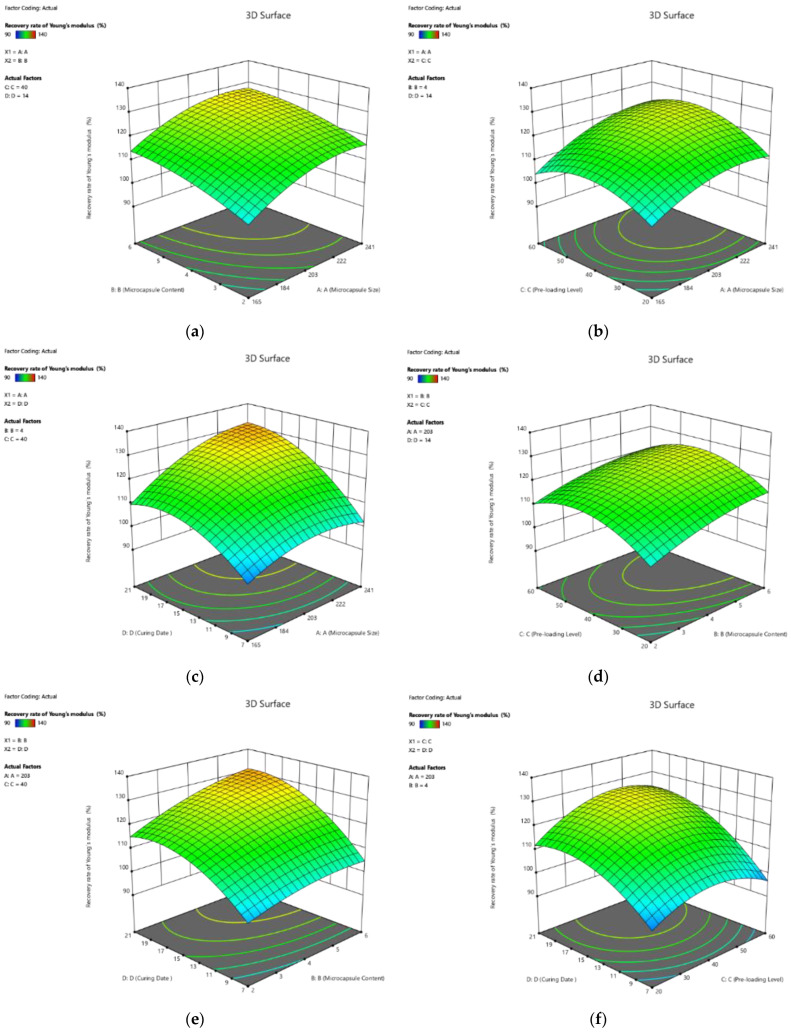
Three-dimension response surface plots of recovery rate of Young’s modulus (Y_4_) in relation to: (**a**) microcapsule size (X_1_) and microcapsule content (X_2_); (**b**) microcapsule size (X_1_) and pre-loading level (X_3_); (**c**) microcapsule size (X_1_) and curing age (X_4_); (**d**) microcapsule content (X_2_) and pre-loading level (X_3_); (**e**) microcapsule content (X_2_) and curing age (X_4_); (**f**) pre-loading level (X_3_) and curing age (X_4_).

The 3D response surface plots based on Equation (11) are shown in Figure 7a–f. Similar to the effect on the healing rate (Figure 6), the recovery rate of Young’s modulus response increased with the increase in the four factors. Moreover, it is obvious that the coefficient of the curing age (10.70) was approximately 3.47 times higher than that of the pre-loading level, indicating that the curing age can significantly affect the recovery rate of the self-healing cement paste. Moreover, the interactions between the two factors provided a limited contribution to the recovery rate, excepting the terms with X_4_ (curing age), which again, confirmed the most significant influence of curing age.

##### Effect on Healing Rate of Peak Strain

**Figure 8:**
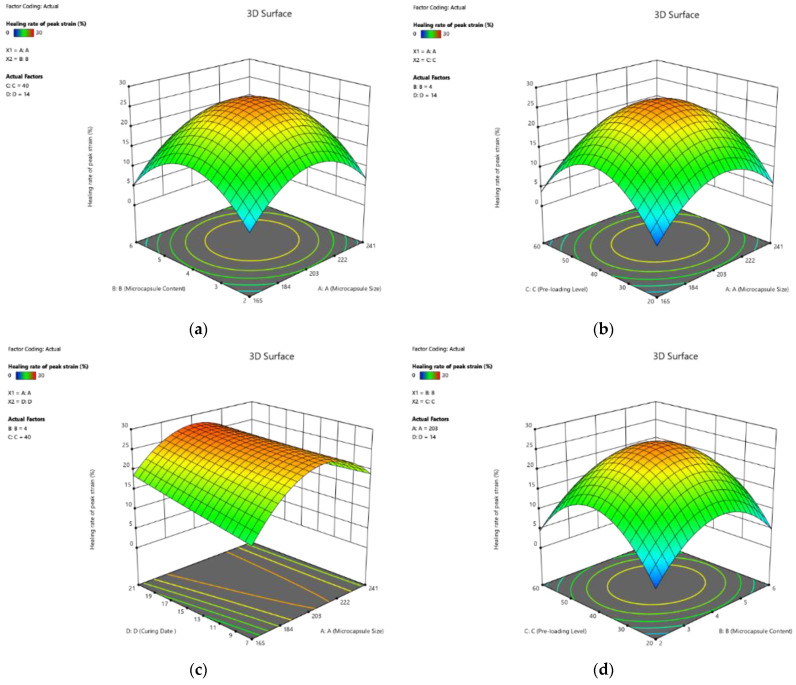

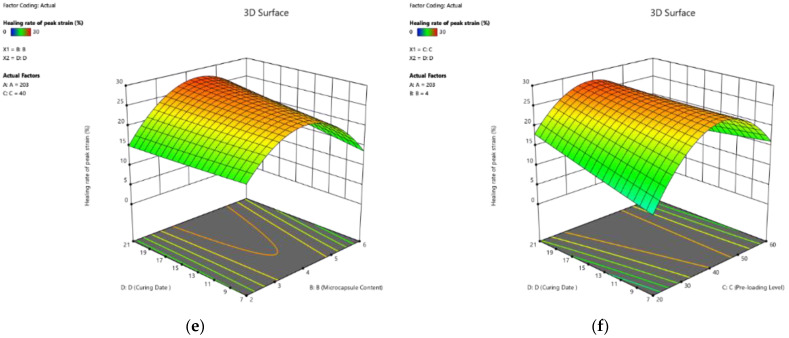
Three-dimension response surface plots of recovery rate of peak strain (Y_5_) in relation to: (**a**) microcapsule size (X_1_) and microcapsule content (X_2_); (**b**) microcapsule size (X_1_) and pre-loading level (X_3_); (**c**) microcapsule size (X_1_) and curing age (X_4_); (**d**) microcapsule content (X_2_) and pre-loading level (X_3_); (**e**) microcapsule content (X_2_) and curing age (X_4_); (**f**) pre-loading level (X_3_) and curing age (X_4_).

The 3D response surface plots based on Equation (12) are shown in Figure 8. It is shown that the four factors positively contributed to the healing rate of peak strain. For example, the coefficients of all the four single factors were positive. Moreover, the value of coefficients revealed that curing age contributes to the healing rate of peak strain more significantly than other factors. It should be noted that the interactions between the factors showed almost equal contributions to the healing of peak strain. For example, the coeffect of X_3_X_4_ was −0.34, which was higher than that of X_4_ (1.38), indicating that the effect of the combined factors could not be ignored.

##### Effect on Recovery Rate of Peak Strain

**Figure 9:**
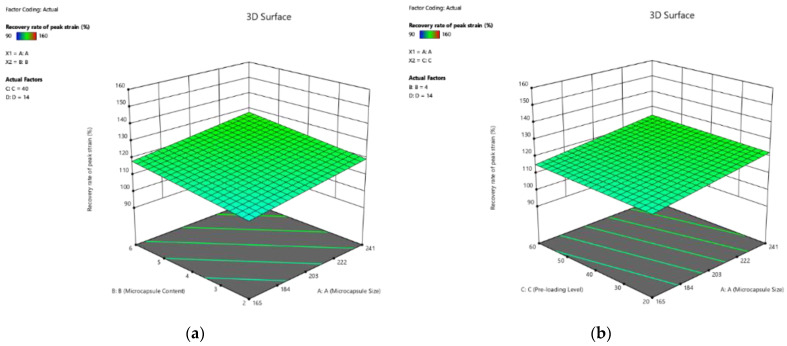

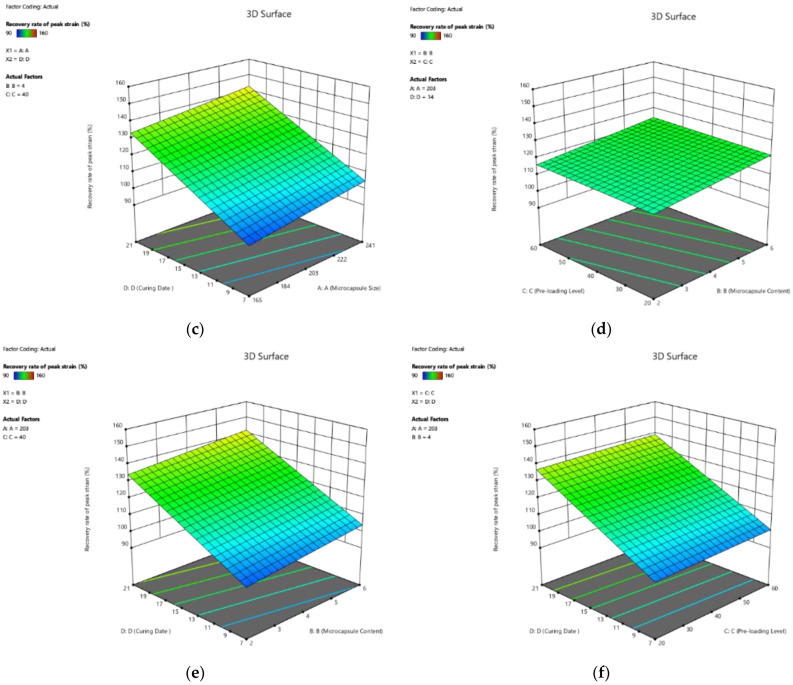
Three-dimension response surface plots of recovery rate of peak strain (Y_6_) in relation to: (**a**) microcapsule size (X_1_) and microcapsule content (X_2_); (**b**) microcapsule size (X_1_) and pre-loading level (X_3_); (**c**) microcapsule size (X_1_) and curing age (X_4_); (**d**) microcapsule content (X_2_) and pre-loading level (X_3_); (**e**) microcapsule content (X_2_) and curing age (X_4_); (**f**) pre-loading level (X_3_) and curing age (X_4_).

The 3D response surface plots, visualising the effect of the four factors on the recovery rate of peak strain of cement paste, are plotted as the response surfaces in Figure 9. As shown in Equation (13), the healing rate of peak strain increased with the increase in the four factors. It should be noted that the influence of curing age was more significant than the other three factors, in terms of microcapsule size, pre-loading level, and microcapsule, which were 4.06, 4.69, and 16.15 times higher, respectively. However, based on the numerical simulation, the interaction between the factors was ignored.

#### 3.3.3. Discussion

To compare the effects of each factor on the healing performance of cementitious material incorporated with PMMA/epoxy microcapsules, the coefficients of all regression models are summarised in Table 7. Since the *p*-value < 0.05 would indicate that the term is significant at 95% confidence, the significant terms for each response in Table 7 are thus bold to be easily identifiable.

As shown in the table, the microcapsule size had mainly affected the recovery rate of all three parameters (peak strength, Young’s modulus, and peak strain) and the healing rate of peak strength and Young’s modulus. The increase in microcapsule size led to an increase in the healing rate and recovery rate. It is well known that the large size of the microcapsule can contain more healing agents. When cementitious material cracks, the large size microcapsules can release more healing agents, which may react with the curing agent and fix the cracks [22]. However, it should be noticed that, although it is a benefit for the healing performance of cement pastes, as presented in Section 3.2, the increment of the microcapsule size may lead to a decrease in the mechanical property of cement pastes. Therefore, depending on the type of microcapsule, the microcapsule size should be confined to a certain range, for example, the range of gelatine/gum. Arabic shell microcapsules containing sodium silicate are normally 250–350 nm for better performance [53].

Similarly, the microcapsule content showed its influence on all six responses except the healing rate of the peak strain. Similar to the effect of microcapsule size, the increased content of microcapsules can provide more curing agent embedding in the cementitious matrix, which consequently, is beneficial for the healing process of the cementitious materials [34]. Nonetheless, as shown in Figure 3, adding more microcapsules decreased the mechanical property of cement pastes. This could be due that the addition of more microcapsules increasing the porosity of the cement pastes [22].

In terms of the pre-loading level, it can be seen that the higher pre-loading shows a higher healing and recovery rate. This is mainly because the higher pre-loading leads to more cracks generated inside the matrix, which can increase the change to trigger the microcapsules [33].

Lastly, the curing age shows the most significant influence on the healing performance of cementitious materials. With increasing the curing age, the improvement in the healing performance of cementitious materials becomes obvious. This could be due that the self-healing performance of epoxy-based microcapsules can be only achieved when the epoxy meets the curing agent. However, due to the nature of the curing agent, the bond strength and stiffness of the cured epoxy gradually increased with curing age [54].

Furthermore, it should be noted that based on statistical analysis the interaction between any two factors, (i.e., X1X2, X3X4) can still affect the self-healing performance of PMMA/epoxy microcapsule-based cementitious materials. Unfortunately, such interactions lead to a more complex situation in analysing the mechanical property of hardened cement pastes. Therefore, further investigation should be conducted to understand the nature of these interactions.

### 3.4. Desirability Functions for Numerical Optimisation

**Figure 10:**
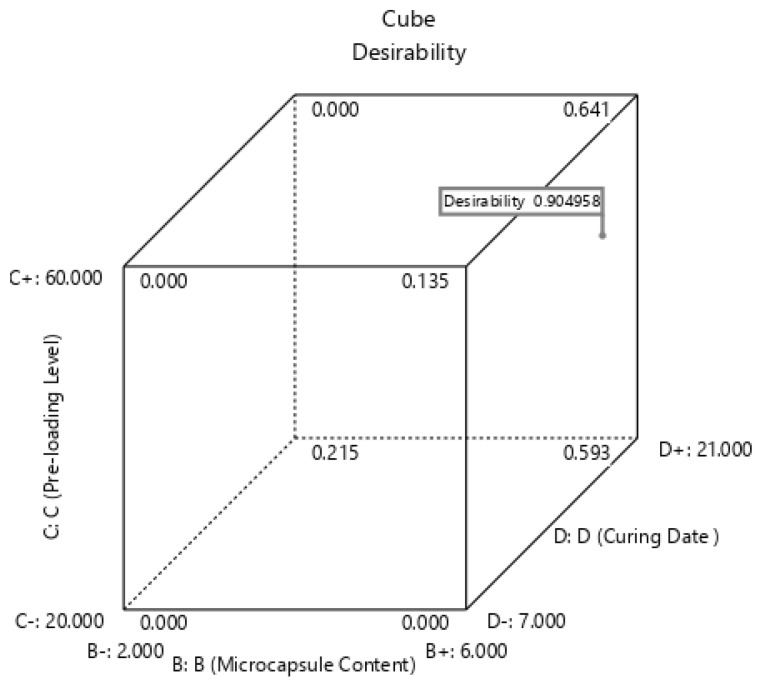
Cubic graphs of the desirability function based on numerical optimisation at 203 nm of microcapsule size (203 nm).

The desirability function (DF) is normally applied to establish the optimum criteria based on multi-variables (factors). The general procedure is to convert the desirability function $Y_{k}$ for each response into the unique desirability function as $d_{k}=f\left(Y_{k}\right)\;\left(0\;\leq \;d_{k}\leq 1\right)$, in which the better optimisation could be achieved when $\;d_{k}$ is close to 1. The numerical optimisation process was conducted, the parameters and predicted target values are listed in Table 8 and cubic graphs of the desirability functions are plotted in Figure 10. It should be noted that since it is very difficult to precisely control the microcapsule size, considering the general trend from results, the microcapsule size was set as 203 nm during the optimisation process. As shown in the table and figure, the predicted values for the six responses generated from the optimisation are 66.62% of the healing rate of peak strength, 152.20% of the recovery rate of peak strength, 44.71% of the healing rate of Young’s modulus, 131.16% of the recovery rate of Young’s modulus, 22.56% of healing rate of peak strain, and 141.50% of the recovery rate of peak strain with the highest desirability of 0.9060. Accordingly, the optimum conditions of the four variables (factors) are 203 nm, 5.59%, 43.56%, and 21 days for the microcapsule size, microcapsule content, pre-loading level, and curing age, respectively. Thus, it can be conducted that by following the optimum recipe computed for the four factors, a PMMA/epoxy microcapsule-based self-healing cementitious material with desirable mechanical properties can be obtained. 

### 3.5. Verification

Three repeating tests were conducted to verify the numerical optimisation process and are presented in Table 9. As shown in the table, the average values obtained were 63.67% for the healing rate of peak strength, 145.22% for a recovery rate of peak strength, 40.34% for the healing rate of Young’s modulus, 132.22% for the recovery rate of Young’s modulus, 27.66% for healing rate of peak strain, and 133.84% for a recovery rate of peak strain, which were 95.71%, 95.42%, 90.22%, 100.81%, 122.62%, and 94.59% of the values predicted by the optimisation, respectively. These verified results were in very good agreement with the predicted value from the generated models.

### 3.6. Pore Structure

**Figure 11:**
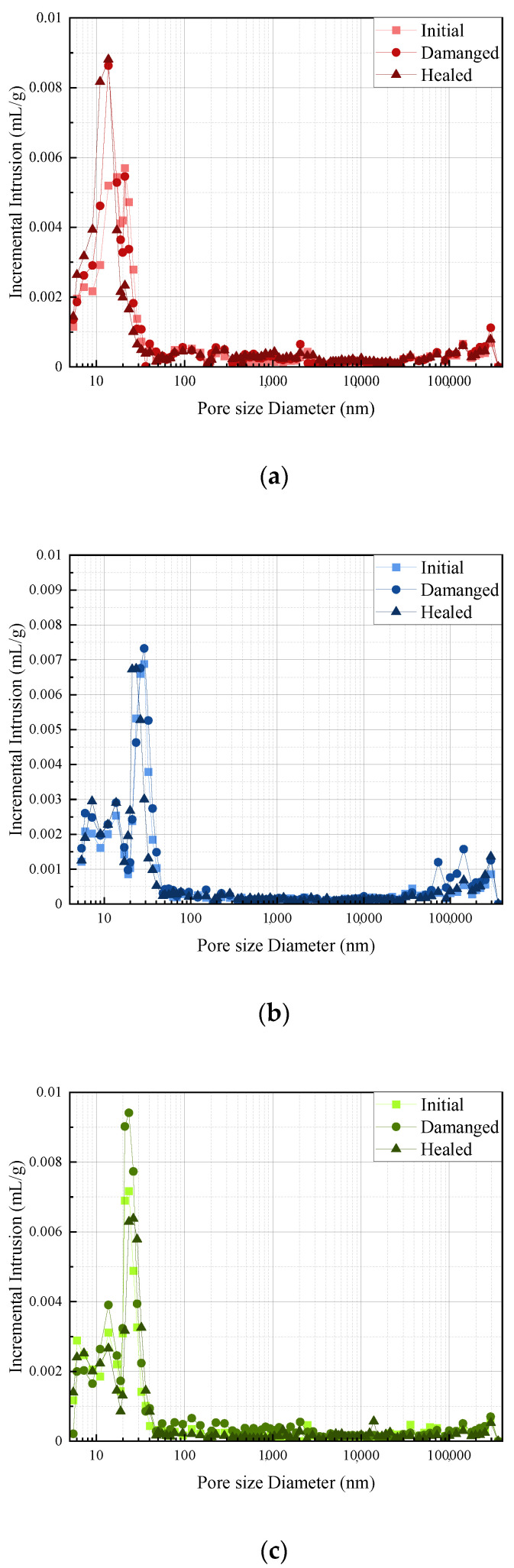
Incremental intrusion of selected self-healing cementitious material: (**a**) specimen of Run 1; (**b**) specimen of Run 15; (**c**) specimen of Verification Run 1.

**Figure 12:**
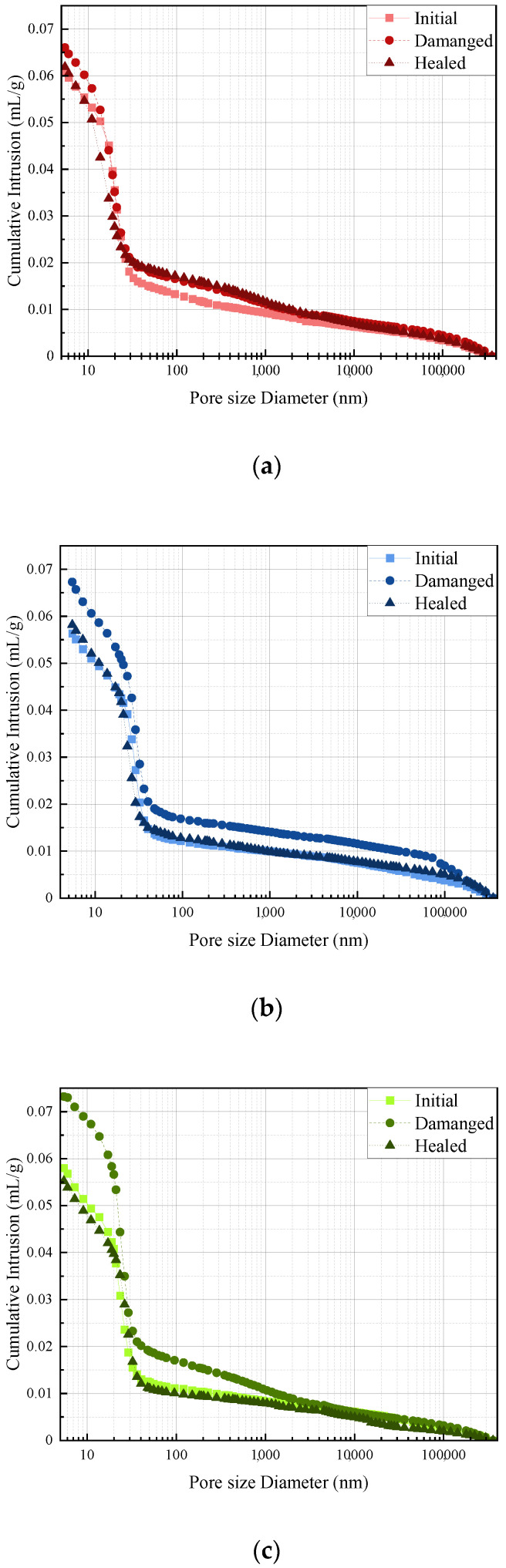
Cumulative intrusion of selected self-healing cementitious material: (**a**) specimen of Run 1; (**b**) specimen of Run 15; (**c**) specimen of Verification Run 1.

The pore structures of three typical mixes, in terms of Run 1, Run 15, and Verification Run 1, in different states (initial, damaged, and healed) were investigated by using MIP, and the details of the results are presented in Figure 11 and Figure 12. In addition, the generated parameters of the pore structure, including in terms of porosity, average pore diameter, pore volume and fractions of gel pore capillary pore, and macropores defined as diameters less than 10 nm, ranging from 10 to 100 nm, and more than 100 nm, respectively [55], are summarised in Table 10. As shown in the figure, for all three samples, compared to the initial state, the porosity, the average pore diameter, and the pore volume were significantly increased when the specimen was in a damaged state, which might be due to the introduced cracks from the pre-loading process. However, after the self-healing process occurred, the porosity average pore diameter and pore volume were further reduced, indicating that the pore structure of the specimens was refined, which further confirmed the improved mechanical property of the specimens as demonstrated in Section 3.3. Moreover, it should be noted that the proportion of the micropores, which is related to the compressive strength (peak strength) of the specimen, was significantly increased when the pre-loading was applied, while it further decreased after the healing process occurred, which further confirmed the results presented in Table 6 and Table 9. This can be attributed to the repairing work of epoxy inside the microcapsule, which can fill macropores, resulting in less porosity and a reduced proportion of microcapsules.

## 4. Conclusions

The influence of the four variables, namely, microcapsule size (X_1_), microcapsule content (X_2_), pre-loading (X_3_), and curing age (X_4_), on the mechanical properties of PMMA/epoxy microcapsule in terms of healing rate of peak strength (Y_1_), the recovery rate of peak strength (Y_2_), the healing rate of Young’s modulus (Y_3_), the recovery rate of Young’s modulus (Y_4_), the healing rate of peak strain (Y_5_), and recovery rate of peak strain (Y_6_) was investigated via BBD and the best healing performance was obtained by numerical optimisation. Based on the results obtained, the following conclusions can be drawn:

- Microcapsule size was identified as the significant factor affecting the healing and recovery rate of peak strength, Young’s modulus, and the peak strain of PMMA/epoxy microcapsule-based self-healing cementitious materials. The microcapsule with large size led to a decrease in the mechanical properties of cementitious material, whereas it increased the healing performance of cement pastes.
- The six parameters were influenced by microcapsule content. The increase in microcapsule content was shown to increase the healing and recovery rates of cementitious materials, but it decreased the initial peak strength, Young’s modulus, and the peak strain of hardened cement.
- Pre-loading level obviously affected the healing performance of microcapsules. With the increase in pre-loading, the healing efficiency decreased.
- The curing age has been identified as the most significant factor affecting the healing performance of PMMA/epoxy microcapsule. The extension of curing age is beneficial for cementitious materials to achieve self-healing performance.
- The numerical optimisation showed that the best healing performance for the six responses was 66.62% for the healing rate of peak strength, 152.20% for the recovery rate of peak strength, 44.71% for the healing rate of Young’s modulus, 131.16% for the recovery rate of Young’s modulus, 22.56% for the healing rate of peak strain, and 141.50% for the recovery rate of peak strain with the desirability of 0.9050. The verification process further confirmed the validation of the predicted value generated from the six response models.
- MIP results further confirmed the healing performance of three selected specimens under initial, damaged, and healed states.

Although the healing performance of PMMA/epoxy microcapsule on the mechanical properties of self-healing cementitious materials was evaluated and discussed in detail, the interactions among the four variables have not been clearly interpreted in the current study, which requires further investigation to understand the mechanism behind. Moreover, the healing process should be tracked from both macro and micro levels, and the healing performance on other properties, in particular, durability, should be further studied.

## Figures and Tables

**Table 1:** Chemical compositions and physical properties of cement (mass, %).

SiO_2_	Al_2_O_3_	CaO		Fe_2_O_3_	MgO	R_2_O	f-CaO	SO_3_		Loss	Cl^−^
20.53	4.45	62.05		3.17	2.81	0.55	0.80	2.10		1.74	0.032
C_3_S			C_2_S			C_3_A			C_4_AF		
57.58			18.55			7.14			11.37		
Fineness%		Specific Surface Area m^2^/kg				Density g/cm^3^		Standard Consistency/%			
0.6		359				3.12		25.80			

**Table 2:** Size distribution and roundness of the microcapsule.

Code	Rotation Speeds/rpm	Particle Size (D50)/µm	Specific Surface Area/m^2^/kg	Roundness
Small	600	164.98	39.97	0.842
Medium	400	202.38	32.91	0.844
Large	200	241.16	27.91	0.834

**Table 3:** Mixing proportion of self-healing cementitious materials.

Code	Cement/g	Water/g	Microcapsule/g			Curing Agent/g	Pre-Loading/%	Curing Age/Days
			Small	Medium	Large			
R	600	174					20%, 40%, 60%	7, 14, 21
S2			12			4.8		
S4			24			9.6		
S6			36			14.4		
M2				12		4.8		
M4				24		9.6		
M6				36		14.4		
L2					12	4.8		
L4					24	9.6		
L6					36	14.4		

**Table 4:** Actual values for the variables used in the experimental design.

Independent Variables	Symbols	Unit	Actual Values for the Coded Values		
			−1	0	+1
Microcapsule Size	X_1_	µm	165	203	241
Microcapsule Content	X_2_	wt%	2	4	6
Pre-Loading	X_3_	%	20	40	60
Curing age	X_4_	Days	7	14	21

**Table 5:** The points for 4 factors BBD design (coded value).

Runs	Microcapsule Size (X_1_)	Microcapsule Content (X_2_)	Pre-Loading (X_3_)	Curing Age (X_4_)
1	0	0	0	0
2	0	−1	1	0
3	1	0	0	1
4	0	0	−1	1
5	1	0	−1	0
6	1	0	1	0
7	1	0	0	−1
8	−1	0	−1	0
9	0	0	1	−1
10	−1	0	0	−1
11	0	0	1	1
12	0	0	−1	−1
13	0	0	0	0
14	−1	1	0	0
15	1	1	0	0
16	1	−1	0	0
17	0	1	−1	0
18	0	0	0	0
19	0	1	1	0
20	0	−1	0	−1
21	0	−1	−1	0
22	0	1	0	1
23	0	0	0	0
24	0	0	0	0
25	−1	0	0	1
26	0	−1	0	1
27	−1	0	1	0
28	0	1	0	−1
29	−1	−1	0	0

**Table 6:** Technical details of the points for the 4-factor BBD design (coded value) and corresponding responses.

Run	Factors				Responses					
	Microcapsule Size	Microcapsule Content	Pre-Loading Level	Curing Age	Healing Rate of Peak Strength	Recovery Rate of Peak Strength	Healing Rate of Young’s Modulus	Recovery Rate of Young’s Modulus	Healing Rate of Peak Strain	Recovery Rate of Peak Strain
1	0	0	0	0	41.64	125.78	35.21	120.07	25.72	121.06
2	0	−1	1	0	19.94	108.07	29.96	109.46	3.61	112.72
3	1	0	0	1	54.83	143.71	48.22	129.99	22.55	153.28
4	0	0	−1	1	36.06	128.98	12.75	110.70	18.39	134.77
5	1	0	−1	0	29.78	122.68	16.57	111.34	2.45	116.91
6	1	0	1	0	37.04	125.17	43.84	120.60	3.37	118.72
7	1	0	0	−1	12.13	104.07	16.70	102.34	22.72	96.73
8	−1	0	−1	0	6.60	98.92	8.02	103.40	3.35	110.95
9	0	0	1	−1	10.20	99.29	9.80	97.64	18.84	99.66
10	−1	0	0	−1	6.34	96.78	5.72	97.37	6.99	100.84
11	0	0	1	1	50.93	135.99	39.04	123.65	14.93	139.22
12	0	0	−1	−1	4.08	98.67	−2.42	95.81	10.16	99.84
13	0	0	0	0	38.26	129.24	37.72	124.33	23.33	126.31
14	−1	1	0	0	41.56	131.36	19.65	111.72	7.76	121.54
15	1	1	0	0	58.77	147.94	44.57	128.45	8.06	128.38
16	1	−1	0	0	20.55	112.49	30.79	118.52	7.42	127.43
17	0	1	−1	0	32.26	123.49	22.39	115.86	4.98	117.38
18	0	0	0	0	43.40	130.10	33.28	126.45	27.49	115.33
19	0	1	1	0	48.85	132.23	39.83	120.75	2.73	123.18
20	0	−1	0	−1	7.33	99.60	8.30	97.39	13.51	100.45
21	0	−1	−1	0	12.45	106.60	9.71	103.04	3.26	107.33
22	0	1	0	1	66.52	152.20	46.08	132.82	20.86	148.29
23	0	0	0	0	42.08	123.42	31.25	118.38	26.46	117.24
24	0	0	0	0	39.16	124.31	38.02	124.36	28.22	127.31
25	−1	0	0	1	31.83	119.97	18.44	109.09	14.28	125.86
26	0	−1	0	1	39.64	129.59	29.66	116.60	12.23	129.92
27	−1	0	1	0	20.78	105.96	19.22	105.01	4.86	107.80
28	0	1	0	−1	21.83	108.19	17.02	103.91	14.40	105.06
29	−1	−1	0	0	11.48	100.93	10.97	101.28	10.18	117.28

**Table 7:** Coefficients of the variables of all responses.

	Healing Rate of Peak Strength (Y1)		Recovery Rate of Peak Strength (Y2)		Healing Rate of Young’s Modulus (Y3)		Recovery Rate of Young’s Modulus (Y4)		Healing Rate of Peak Strain (Y5)		Recovery Rate of Peak Strain (Y6)	
	Coefficient	*p*-Value	Coefficient	*p*-Value	Coefficient	*p*-Value	Coefficient	*p*-Value	Coefficient	*p*-Value	Coefficient	*p*-Value
Intercept	40.91		126.57		35.10		122.72		26.24		118.99	
X_1_	7.88	<0.0001	8.51	<0.0001	9.89	<0.0001	6.95	<0.0001	1.60	0.1492	4.76	0.0093
X_2_	13.20	<0.0001	11.51	<0.0001	5.85	<0.0001	5.60	<0.0001	0.72	0.5051	4.06	0.0240
X_3_	5.54	<0.0001	2.28	0.0937	9.56	<0.0001	3.08	0.0004	0.48	0.6543	1.18	0.4916
X4	18.16	<0.0001	16.99	<0.0001	11.59	<0.0001	10.70	<0.0001	1.38	0.2070	19.06	<0.0001
X_1×2_	2.04	0.2555	1.25	0.5778	1.27	0.3772	−0.13	0.9137	0.77	0.6789		
X_1×3_	−1.73	0.3312	−1.14	0.6128	4.02	0.0123	1.91	0.1231	−0.15	0.9363		
X_1×4_	4.31	0.0253	4.11	0.0824	4.70	0.0047	3.98	0.0042	−1.86	0.3208		
X_2×3_	2.27	0.2071	1.82	0.4214	−0.70	0.6229	−0.38	0.7485	−0.65	0.7249		
X_2×4_	3.09	0.0935	3.51	0.1329	1.93	0.1898	2.42	0.0562	1.94	0.3034		
X_3×4_	2.19	0.2236	1.60	0.4793	3.52	0.0246	2.78	0.0317	−3.04	0.1160		
X_1_^2^	−7.40	<0.0001	−5.08	0.0107	−4.91	0.0005	−5.08	<0.0001	−9.37	<0.0001		
X_2_^2^	−1.22	0.3832	0.26	0.8813	−1.63	0.1589	−2.50	0.0162	−9.99	<0.0001		
X_3_^2^	−10.24	<0.0001	−8.07	0.0004	−9.16	<0.0001	−7.82	<0.0001	−12.25	<0.0001		
X_4_^2^	−6.15	0.0005	−4.19	0.0291	−9.09	<0.0001	−7.81	<0.0001	0.12	0.9365		

Note: X_1_ stands for microcapsule size; X_2_ for microcapsule content; X_3_ for pre-loading level; X_4_ for curing age, X_i_X_j_ for interaction between factor i and factor.

**Table 8:** Characteristics of numerical optimisation.

Parameters	Importance	Weight	Goal	Predict Value
Microcapsule size (X_1_)/nm	3	1	Equal to	203
Microcapsule content (X_2_)/%	3	1	In range	5.59
Pre-loading level (X_3_)/%	3	1	In range	43.56
Curing age (X_4_)/Day	3	1	In range	21
Healing rate of peak strength/%	5	1	Maximise	66.52
Recovery rate of peak strength/%	5	1	Maximise	152.20
Healing rate of Young’s Modulus/%	5	1	Maximise	44.71
Recovery rate of Young’s Modulus/%	5	1	Maximise	131.16
Healing rate of peak strain/%	5	1	Maximise	22.56
Recovery rate of peak strain/%	5	1	Maximise	141.50
Desirability			0.9050	

**Table 9:** Verification for the numerical optimisation.

Parameters	Predict Value	Verification				Relative Error
		Run 1	Run 2	Run 3	Average	
Healing rate of peak strength/%	66.52	67.53	64.83	58.64	63.67	95.71
Recovery rate of peak strength/%	152.20	147.67	147.95	140.05	145.22	95.42
Healing rate of Young’s Modulus/%	44.71	45.39	39.47	36.14	40.34	90.22
Recovery rate of Young’s Modulus/%	131.16	138.64	131.72	126.32	132.22	100.81
Healing rate of peak strain/%	22.56	29.71	23.16	30.13	27.66	122.62
Recovery rate of peak strain/%	141.50	137.76	127.32	136.44	133.84	94.59

**Table 10:** Pore structure of PMMA/epoxy microcapsule-based cementitious materials.

Type	State	Porosity (%)	Average Pore Diameter (nm)	Pore Volume (mL/g)	Pore Size Distribution (%)		
					<10 nm	10~100 nm	>100 nm
Run 1	Initial	12.01	18.23	0.0619	8.88	78.12	13.00
	Damaged	12.48	20.13	0.0660	8.83	74.94	16.23
	Healed	11.95	20.47	0.0608	11.71	72.21	16.08
Run 15	Initial	11.68	21.44	0.5082	10.49	78.01	11.50
	Damaged	13.26	23.81	0.0673	9.94	74.89	15.17
	Healed	11.38	23.13	0.0563	9.45	78.44	12.11
Veri-Run 1	Initial	11.52	20.41	0.0580	11.28	80.73	7.99
	Damaged	13.86	24.72	0.0732	5.79	76.70	17.51
	Healed	10.87	20.83	0.0552	11.44	81.79	6.77

## Data Availability

All the data associated with this study are available from the corresponding author upon request.
